# Prediction and Identification of GPCRs Targeting for Drug Repurposing in Osteosarcoma

**DOI:** 10.3389/fonc.2022.828849

**Published:** 2022-04-07

**Authors:** Manli Tan, Shangzhi Gao, Xiao Ru, Maolin He, Jinmin Zhao, Li Zheng

**Affiliations:** ^1^ Guangxi Engineering Center in Biomedical Materials for Tissue and Organ Regeneration, The First Affiliated Hospital of Guangxi Medical University, Nanning, China; ^2^ Collaborative Innovation Center of Regenerative Medicine and Medical Biological Resources Development and Application of Guangxi Medical University, Nanning, China; ^3^ Department of Orthopaedics Trauma and Hand Surgery, The First Affiliated Hospital of Guangxi Medical University, Nanning, China; ^4^ Guangxi Key Laboratory of Regenerative Medicine, The First Affiliated Hospital of Guangxi Medical University, Nanning, China

**Keywords:** bioinformatics analysis, osteosarcoma, G protein-coupled receptors (GPCRs), risk model, drug targets

## Abstract

**Background:**

Osteosarcoma (OS) is a malignant bone tumor common in children and adolescents. The 5-year survival rate is only 67-69% and there is an urgent need to explore novel drugs effective for the OS. G protein-coupled receptors (GPCRs) are the common drug targets and have been found to be associated with the OS, but have been seldom used in OS.

**Methods:**

The GPCRs were obtained from GPCRdb, and the GPCRs expression profile of the OS was downloaded from the UCSC Xena platform including clinical data. 10-GPCRs model signatures related to OS risk were identified by risk model analysis with R software. The predictive ability and pathological association of the signatures in OS were explored by bio-informatics analysis. The therapeutic effect of the target was investigated, followed by the investigation of the targeting drug by the colony formation experiment were.

**Results:**

We screened out 10 representative GPCRs from 50 GPCRs related to OS risk and established a 10-GPCRs prognostic model (with CCR4, HCRTR2, DRD2, HTR1A, GPR158, and GPR3 as protective factors, and HTR1E, OPN3, GRM4, and GPR144 as risk factors). We found that the low-risk group of the model was significantly associated with the higher survival probability, with the area under the curve (AUC) of the ROC greater than 0.9, conforming with the model. Moreover, both risk-score and metastasis were the independent risk factor of the OS, and the risk score was positively associated with the metastatic. Importantly, the CD8 T-cells were more aggregated in the low-risk group, in line with the predict survival rate of the model. Finally, we found that DRD2 was a novel target with approved drugs (cabergoline and bromocriptine), and preliminarily proved the therapeutic effects of the drugs on OS. These novel findings might facilitate the development of OS drugs.

**Conclusion:**

This study offers a satisfactory 10-GPCRs model signature to predict the OS prognostic, and based on the model signature, candidate targets with approved drugs were provided.

## Introduction

Osteosarcoma is a malignant bone tumor that is common in children and adolescents. Surprisingly, there has been little improvement in the survival rate of patients since the 1970s, with a 5-year survival rate of only 67-69%, which is one of the highest mortality rates among childhood and adolescent cancers (1). Patients with metastases and relapses can hardly be cured by conventional methods like surgery and radiotherapy (2). As an alternative, chemotherapy is also limited by cytotoxicity, drug resistance, etc., with unsatisfactory survival rate and sometimes serious side effects (3, 4), for example, methotrexate-induced acute encephalopathy (5). This may be due to the lack of tissue specificity of chemotherapeutic. Therefore, there is an urgent need to explore novel drugs specifically targeting OS.

Recently, the critical role of GPCRs in bone development, remodeling, and disease has been verified, with 92 GPCRs associated with bone disease and dysfunction (6). The GPCRs are the largest family of transmembrane proteins involved in multiple biological processes, including bone development and remodeling (7, 8), inflammation/immune response (9), tumor growth, and metastasis (10), etc. A growing body of evidence suggests that GPCRs serve as pro-tumor (GPR56 (11), GPR110 (12), PTHR1 (13), PAR1 (14), S1PR3 (15)) or anti-tumor (GPER1 (16), A3AR (17)) in OS. However, most studies only focused on one type of GPCRs, which may not reflect the real condition since the receptors synergistically exert the pro-tumor or anti-tumor effects by highly integrated interactions of numerous receptor signals. Thus, it is imperative to integrate all the GPCRs to investigate their correlation with OS-risk for the screening of the representative GPCRs to predict the OS-risk. Specifically, GPCRs have easy-to-target ligand-binding domains to bind with a variety of chemical regulators, which constitute the most important class of drug targets (18, 19). This may benefit the selection of the appropriate drug for OS therapy by repurposing the known drugs for new indications (20). In this study, in an attempt to find the suitable known-drug repurposing for OS, we investigated the association of the GPCRs with OS progression and determined the GPCRs that serve as drug targets by risk evaluation. Firstly, we analyzed the 85 samples of OS from the TARGET database and assessed the risk correlation of all GPCRs in the GPCRdb with OS. GPCRdb is a database containing all human non-olfactory GPCRs, including over 2000 approved drugs and in-trial agents and nearly 200,000 ligands with activity and availability data (21). Second, to avoid excessive intervention for low-risk patients, we established a risk-score model by using risk-GPCRs and explored the predictive ability of the model and its components on OS risk. We also investigated the correlation between the model and the infiltration abundance of immune cells. Finally, we preliminarily verified the effects of the GPCRs-targeting drugs on OS by using two types of OS cell lines. This research will provide a novel insight into OS therapy.

## Materials and Methods

### Data Source and Preprocessing

Human non-olfactory GPCRs were obtained from GPCRdb (https://gpcrdb.org/), and a total of 395 GPCRs were obtained. Eighty-five OS samples containing 395 GPCRs expression data and clinical information were downloaded from the GDC TARGET on the UCSC XENA platform (22) (https://xena.ucsc.edu/). The single-cell RNA-seq dataset (GSE152048) was downloaded from the GEO database (www.ncbi.nlm.nih.gov/geo).

We preprocessed the TARGET-OS dataset to filter low-expressed GPCRs, the deletion measure was to remove those GPCRs with expression level < 1 and accounting for more than 50% of all samples. Due to the low expression, only 333 GPCRs were finally included in the analysis. The details of these GPCRs and the clinical characteristics of the samples were shown in **Figure 2A**.

### Identification of Key GPCRs and Construction of Prognostic Models

Univariate Cox analysis of the cohort. Perform univariate Cox proportional hazard regression for each GPCRs in the cohort, and performed the “coxph” function of the “survival” package in R with P < 0.05 as the filter threshold.

LASSO-Cox analysis. In order to decrease the number of GPCRs in the risk model, we performed Least Absolute Shrinkage and Selection Operator (LASSO) regression on the GPCRs obtained by univariate Cox analysis to reduce the over-fitting phenomenon. This was realized by performed “glmnet” and “cv.glmnet” functions of the “glmnet” package in R.

Multivariate Cox analysis of risk GPCRs. In order to screen out GPCRs that can collaboratively predict OS risk, and to further simplify the number of GPCRs in the risk model, multivariate Cox analysis was performed on the GPCRs obtained from the LASSO-Cox analysis, and the “Akaike Information Criteria (AIC)” was used for stepwise reduction of the variables. The GPCRs combination with the lowest AIC score was used to further construct a risk model. The receiver operator characteristic (ROC) curve was used to evaluate the accuracy of the model.

### Predict Survival Rate

Kaplan Meier Plotter (23) (http://kmplot.com/analysis/index.php?p=service&cancer=custom_plot) was used to predict the survival rate of the risk model and its genes. Auto-select best cutoff.

### Calculation of TME Immune Cell Infiltration Abundance

The infiltration of the tumor microenvironment (TME) immune cells may important to the patient’s outcome. The CIBERSORTx algorithm (https://cibersortx.stanford.edu/) was used to quantify the infiltration abundance of 22 types of immune cells in OS. The CIBERSORTx parameters were as follows: the input matrix was the RNA-Seq data containing 85 samples and 33464 genes, the 22 immune cell types from Newman et al. (24) were input as the reference of genes signature, 500 times for permutation test, and RNA-seq data without quantile normalization.

#### Calculating the EMT Score

We obtained 77 marker genes of epithelial-to-mesenchymal transition (EMT) from Mak (25). The EMT score for each sample was calculated as $\Sigma_{i}^{N}\frac{M^{i}}{N}-\Sigma_{j}^{n}\frac{E^{j}}{n}$ , like the described in Chen’s study (26), where M represents the expression of the mesenchymal genes, E represents the expression of the epithelial genes, N and n represents the number of mesenchymal genes and epithelial genes respectively.

### Mapping Risk GPCRs to the OS Single-Cell Atlas

In order to detect and evaluate the expression of risk GPCRs in the OS micro-environment, we used the OS single-cell atlas. The “Seurat” package (27) in R was used for the single-cell atlas analysis of the GSE152048 dataset (28). We preprocessed the GSE152048 dataset to filter the low-quality cells and genes, the inclusion criteria for genes were expressed in at least 5 cells, the inclusion criteria for cells were that at least 300 genes were expressed in the cell, and the proportion of mitochondrial genes and hemoglobin genes in the cell were less than 5%. The biomarkers provided in the original article (28) were used for the annotation of the cell population.

### Mining the Targeted Drugs of Risk-Relation-GPCRs

We searched the targeted drugs of GPCRs in the drug&ligands menu of GPCRdb. The screening criteria of the targeted drugs were those have reported in tumor treatment.

### Cell Culture

The osteosarcoma cell line (143B, ATCC: CRL-8303, and HOS, ATCC: HTB-96TM, were purchased from the American Type Culture Collection) were cultured in DMEM/F-12 medium (DMEM, Gibco, Shanghai, China) containing 10% fetal bovine serum (Gibco, American), and 100 µg/mL streptomycin and 100 U/mL penicillin (Solarbio, Beijing, China). All the cells were placed in an incubator containing humidified air with 5% carbon dioxide at 37°C, and the medium was replaced with fresh medium once every 3 days.

### CCK8-Kit to Detect Cell Viability

143B and HOS cells suspension were plated into 96-well plates, the number of cells in each well was about 8×10 ^3^, and they were treated with cabergoline and bromocriptine (0, 1, 10, 50, 100, 150, 200 μmol/L), after 24 hours of incubation, remove the drug, add 100 μl of culture medium and 10μl of CCK-8 detection reagent, and then incubated in an incubator for two hours. Detect the absorbance of each well of the plate in the microplate reader, and set the wavelength to 450 nm. The experiments were repeated three times under the same conditions.

### Colony Formation Experiment

Cells (2×10^3^) of 143B and HOS cells were plated into 6-well plate. After 24 hours, 143B cells 100 μM cabergoline and bromocriptine, and HOS cells were treated with 50-100 μM cabergoline and bromocriptine, 7days later, it was washed twice with phosphate buffer solution and fixed with 4% paraformaldehyde for 10 minutes. After washing with deionized water, it was stained with crystal violet dye for 10 minutes. After washing again, the cell colonies were counted with Image J.

### Statistical Analysis

In this study, R software (version 4.0.3) was applied to the statistical analysis process, unless specifically stated, otherwise, only when p-value < 0.05 was considered statistically significant (main packages include: survival, glmnet, Survminer, survivorROC, Seurat, limma, ggplot2, clusterProfiler, org.Hs.eg. db).

## Result

**Figure 1:**
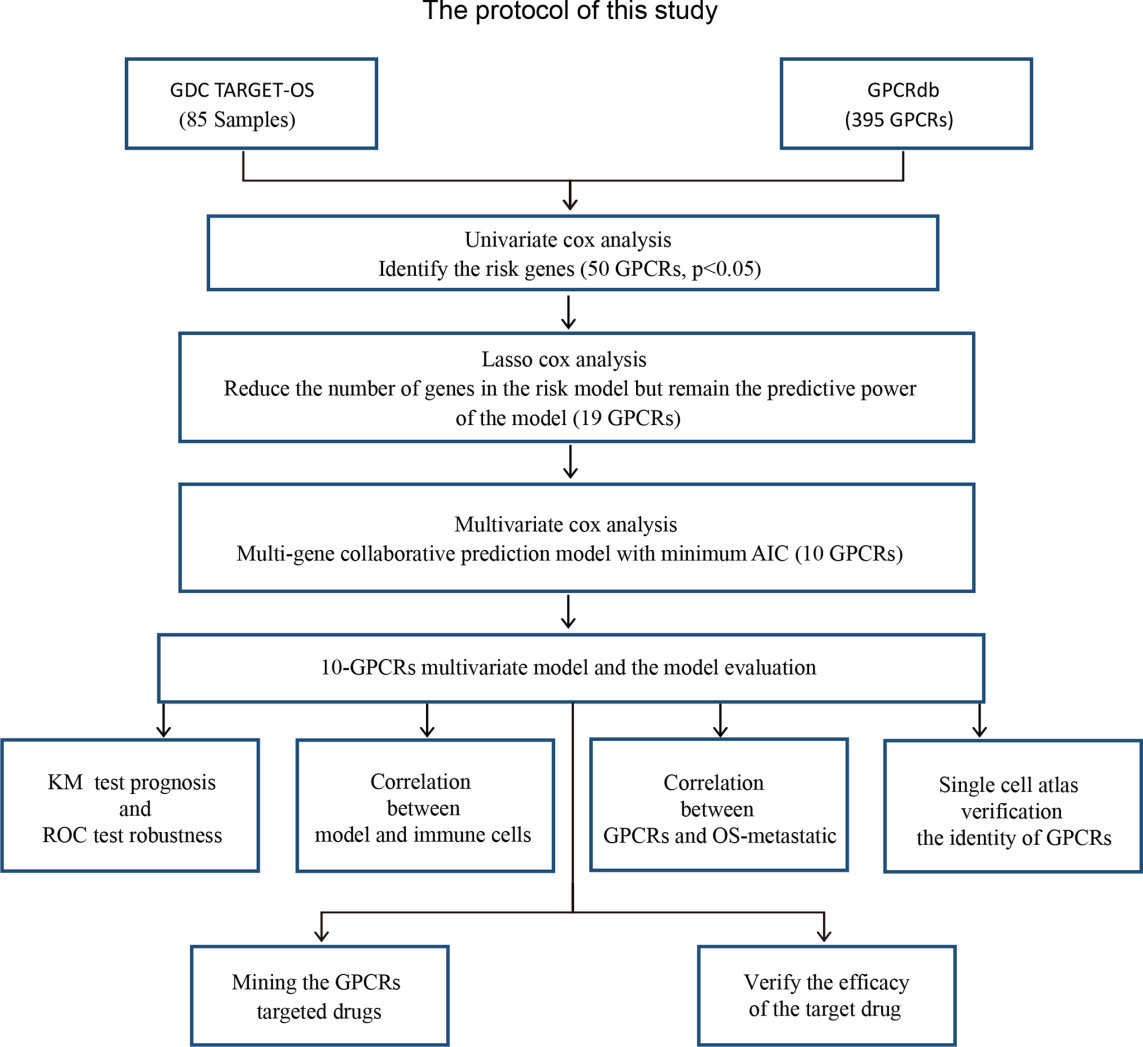
The research protocol for the identification of OS risk-related GPCRs and discovery of their target drugs. GDC TARGET: “Genomic Data Commons, Therapeutically Applicable Research To Generate Effective Treatments”, GPCRdb, G protein-coupled receptor database; AIC, Akaike information criterion; KM, Kaplan–Meier curve.

A protocol was designed to construct a multivariate model for predicting the OS prognosis and to develop targets and targeted drugs. The analysis process was carried out by following protocol (**Figure 1**).

### Identification and Functional Annotation of Risk-Related GPCRs

**Figure 2:**
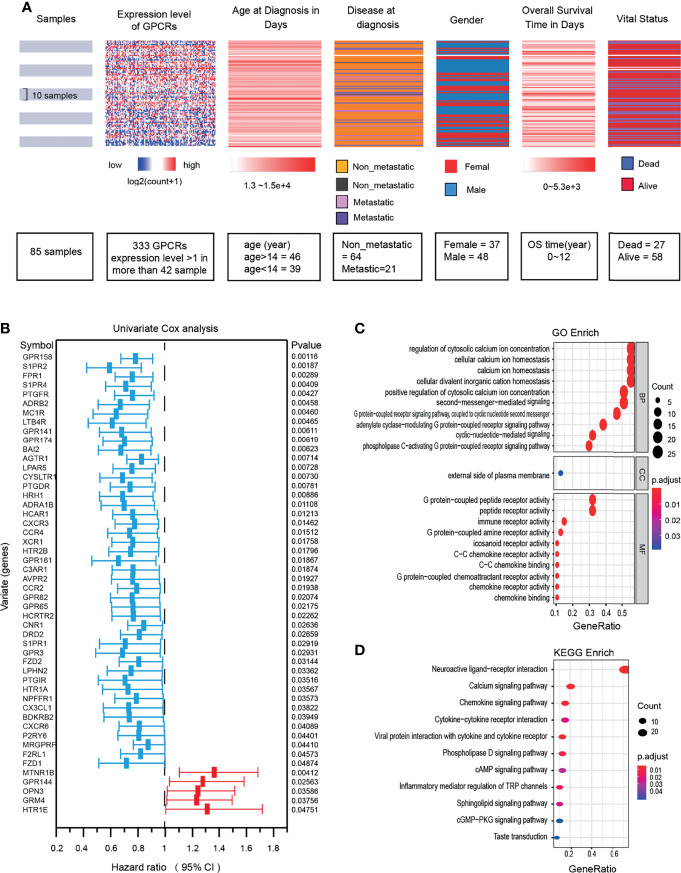
**(A)** OS clinical information and expression levels of GPCRs. **(B)** The forest plot shows the results of univariate Cox regression analysis, there are 50 GPCRs related to OS risk. Hazard ratio <1 indicates the protection factor (blue), Hazard ratio >1 indicates the risk factor (red), CI, Confidence interval. **(C)** Functional enrichment analysis of GPCRs in GO terms. **(D)** Signaling pathway enrichment analysis of GPCRs in the KEGG.

The information of the GPCRs and the OS samples in this study were shown in (**Figure 2A**). 50 GPCRs related to OS risk were obtained through univariate Cox proportional hazard regression analysis (P < 0.05). Among them, the high expression of 45 GPCRs was associated with the OS-low risk (protective factors), while 5 GPCRs’ high expression was associated with the OS-high risk (risk factors) (**Figure 2B**). specified, the GPR158 was the most significant protective factor, and MTNR1B was the most significant risk factor. GO (gene ontology) and KEGG (Kyoto Encyclopedia of Genes and Genomes) enrichment analysis was used to annotate the biological processes, molecular functions, and signaling pathways of the 50 GPCRs. The results showed (**Figures 2C, D**) that, the most enriched GO term were those who participated in the regulation of cytosolic calcium ion concentration, G protein-coupled peptide receptor activity, and immune receptor activity. In terms of KEGG, neuroactive ligand−receptor interaction, calcium signaling pathway, and chemokine signaling pathway were the most enrichment signaling pathways for risk-related GPCRs.

### Construction of Risk Prognosis Model Based on GPCRs

**Figure 3:**
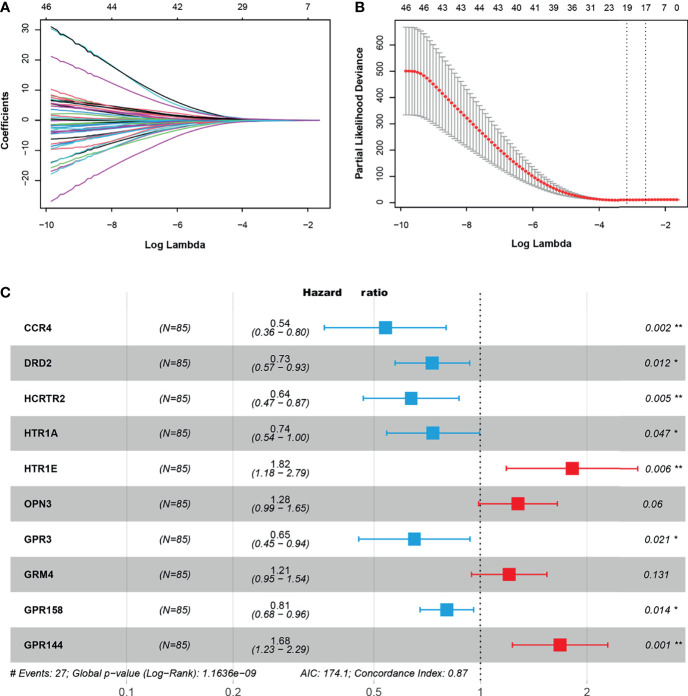
**(A)** The trajectory of independent variables, the horizontal axis, and the vertical axis represent the logarithm of the independent variable lambda and the coefficient of the independent variable respectively. **(B)** The confidence interval under each lambda. **(C)** The key GPCRs used to build a risk model, hazard ratio (HR) < 0 indicated the hazard reduction, HR > 0 indicated the hazard increase. “*” represents 0.01 < p < 0.05, “**” represents 0.001 < p < 0.01.

The 50 GPCRs were considered to be numerous prognostic factors and the workload was still heavy in screening the targets. Therefore, we performed LASSO-Cox regression analysis to decrease the numbers of GPCRs, meanwhile reducing the over-fitting phenomenon. The results show that with the increase of Lambda, the coefficient of the independent variables was decreasing, and the number of independent variables whose coefficient’ absolute value was greater than 0 was decreasing (**Figure 3A**). When using 10-fold cross-validation to construct the model, we could see when the cross-validation was the smallest, only 19 GPCRs remained as candidate GPCRs for subsequent analysis (**Figure 3B**). To further decrease the number of GPCRs, 19 GPCRs were performed multivariate Cox regression analysis and AIC stepwise regression analysis. There is a consensus that when the AIC is the smallest, the model is the best, it gave the model sufficient fit with fewest variables. Finally, the fewest of GPCRs were obtained to construct the model (**Figure 3C**), they were: CCR4, DRD2, HCRTR2, HTR1A, HTR1E, OPN3, GPR3, GRM4, GPR158, GPR144. Together, these 10 GPCRs would be used to build the final prognostic model.

**Figure 4:**
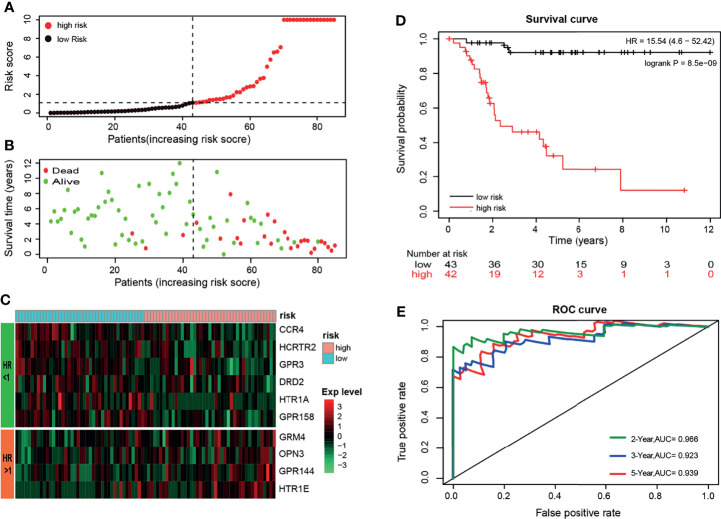
**(A)** Sort the patients by risk-score, black’ dot and red’ dot represent the low-risk group and high-risk group respectively, and the dashed line represents the median risk score. **(B)** Sort the patients by risk score, green and red represent alive and dead respectively, and the dashed line represents the median risk score. **(C)** The heat map shows the expression levels of the 10-GPCRs. **(D)** Kaplan-Meier survival curve distribution of the OS patients (p < 0.001). **(E)** ROC curve of the model and the green, blue, red curve represents the rate of correct prediction in 2, 3, 5 years.

The 10 GPCRs were integrated as a risk prognosis model to evaluate the prognostic value of the OS patients. First, integrate these GPCRs into the multivariate Cox model, and the “predict” function of the “survival” package in R was used to calculate the risk score of each OS patient. Then, according to the median risk score, the OS patients were divided into high-risk and low-risk groups. The risk curves and scatter plots was illustrated the relation of the model’s risk score and the vital status of OS patients (**Figures 4A, B**), we could see that the higher of the risk score, the more of the dead patients. The heat map showed the relationship between the expression level of 10 GPCRs and the risk score (**Figure 4C**). Among them, 6 GPCRs with HR < 1 (CCR4, HCRTR2, GPR3, DRD2, HTR1A, GPR158), and their high expression was related to the low-risk. The 4 GPCRs with HR>1 (GRM4, OPN3, GPR144, HTR1E), and their high expression was related to the high-risk. The KM survival curve of the patient suggested that the survival probability of the OS patients in the low risk-score group was significant prolonged (p=8.5e-9) than those in the high risk-score group, which indicated that the risk score of the model has a satisfactory prognostic value (**Figure 4D**). In addition, the R package “survivalROC” was used to calculate the area under the ROC curve to quantify the prediction accuracy of the model (**Figure 4E**). The results showed a satisfactory prognostic value of the model in the existing samples, it could accurately predict the survival rate of patients in 2, 3, and 5 years (with AUC > 0.9).

**Figure 5:**
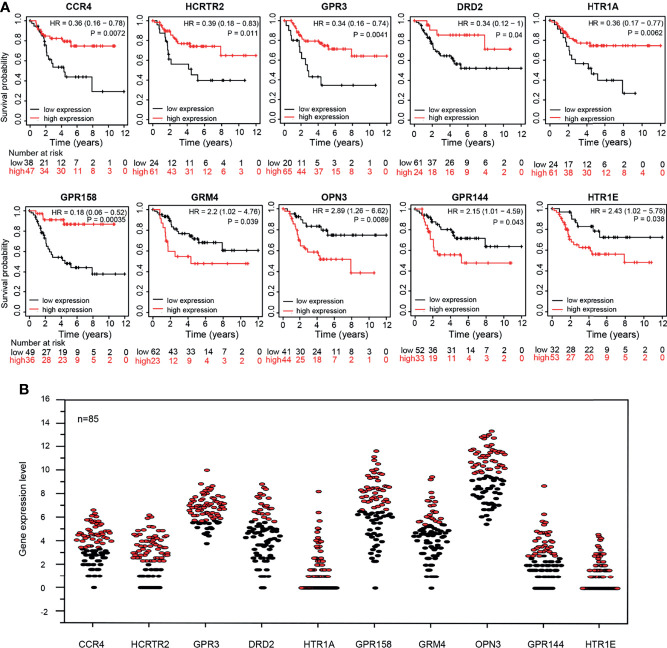
**(A)** Kaplan-Meier survival curve of 10 GPCRs expression level and patient survival rate. **(B)** The expression levels of 10 GPCRs in the OS sample, red and black represent the high expression group and the low expression group respectively.

The KM survival curves of 10 GPCRs were shown in (**Figure 5A**), and all of 10 GPCRs have a significant prognostic value (P <0.05). The grouping of samples was performed by Kaplan-Meier plotter according to GPCRs expression auto select best cutoff (**Figure 5B**).

### Univariate and Multivariate Cox Analysis of the Model and Clinical Features

The independence of 10-GPCRs signature models in clinical applications was tested by using univariate and multivariate Cox regression analyses (**Table 1**). Using the binary classification method, the samples were divided into two groups for analysis according to metastatic (yes VS. no), gender (male VS. female), age (>14 VS. <14), and risk score (high VS. low). The univariate analysis results suggested that the risk-score of the model was significant associated with the hazard ratio (p = 9.81e-06). At the same time, the results of the multivariate analysis indicated that the risk score could be used as an independent factor because it was not interfered with by other factors (hazard ratio=15.627, 95% confidence interval = 4.364 - 55.956, p = 2.40e-05). Similarly, here we found that metastatic was also an independent prognostic factor.

**Table 1:** Univariable analysis and multivariable analysis of the model and clinical features.

	Univariable analysis				Multivariable analysis			
Variables	95% CI of HR				95% CI of HR			
	HR	Lower	Upper	p	HR	Lower	Upper	p
Metastatic (Yes & No)	4.764	2.221	10.22	6.10e-05	2.864	1.222	6.713	0.015
Gender (Male & Femal)	0.713	0.335	1.521	0.382	0.914	0.399	2.092	0.832
Age ( >14 & < 14)	0.757	0.356	1.611	0.470	0.549	0.249	1.212	0.138
Risk (high & low)	15.536	4.605	52.417	9.81e-06	15.627	4.364	55.956	2.40e-05

### The Correlation Between the 10 GPCRs and Metastasis

**Figure 6:**
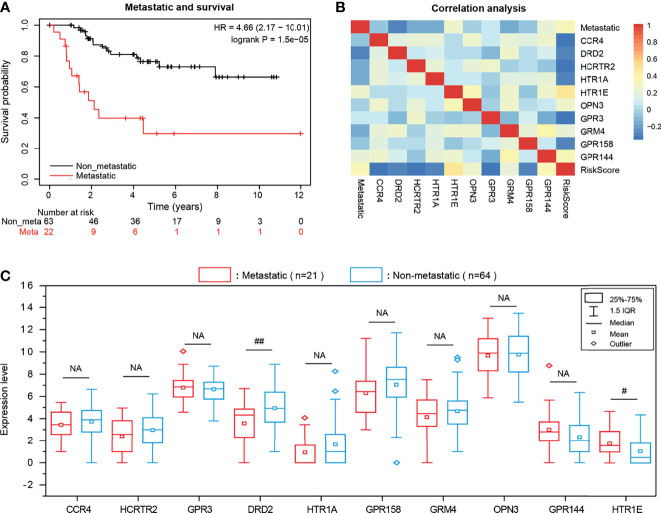
**(A)** KM survival curve of OS metastasis and patient survival rate. **(B)** The Pearson correlation among the expression of 10 GPCRs and OS metastatic and risk scores. **(C)** The expression level of risk genes in OS metastatic and non-metastatic samples, NA represents no significance, “# “represents 0.01 <P < 0.05, “##” represents 0.001 <P < 0.01.

Metastasis is generally considered to be an intractable feature of the OS. Here the KM survival curve showed that the survival rate of non-metastatic patients were significantly prolonged than those of metastatic patients (P = 1.5e-05), confirming the metastasis has a useful prognostic value for OS (**Figure 6A**). Based on this result, we also explored the Pearson correlation between the expression of 10-GPCRs and OS-metastasis and found that DRD2 was the GPCRs that most negatively correlated with OS-metastasis, indicating that the higher expression of DRD2, the lower rate of metastasis (**Figure 6B**). To further reveal the expression of 10 GPCRs in metastatic-OS and non-metastatic-OS (**Figure 6C**), we analyzed the expression of 10 GPCRs in both type OS, and the results showed that the expression of DDR2 in non-metastatic-OS was significantly higher than the metastatic-OS (P = 0.0031), while the expression of HTR1E in metastatic-OS was significantly higher than the non-metastatic-OS (P = 0.018). In addition, it’s may note that the expression of GPR158 in non-metastatic-OS was seemed higher than the metastatic-OS (P = 0.057, close to significance). There was no significant difference in the expression of other GPCRs in metastatic-OS and non-metastatic-OS.

### The Association Between 10-GPCRs Model and TME-Infiltrating Immune Cells

**Figure 7:**
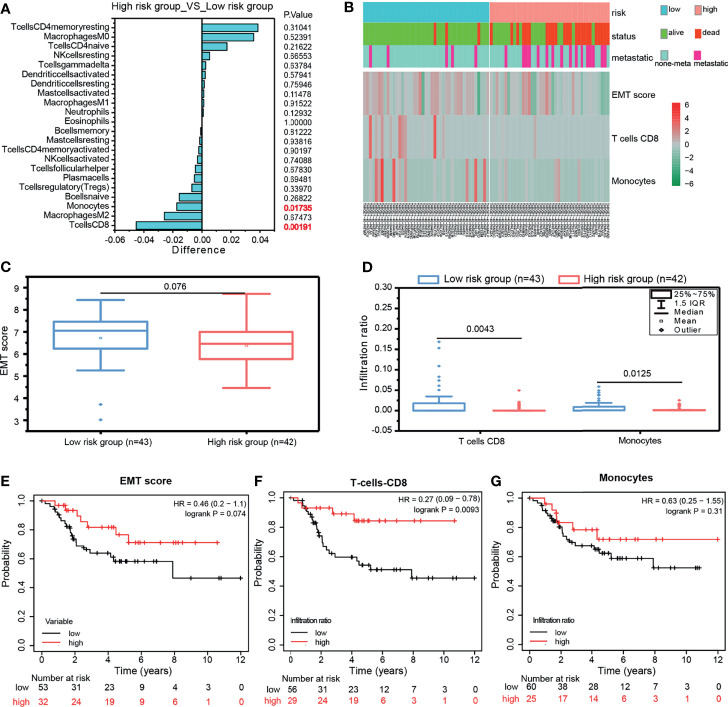
**(A)** Compare the infiltration abundance of immune cells between the high-risk group and low-risk group of the 10-GPCRs model, Differences < 0 indicates that the immune cells were more enriched in the low-risk group. **(B)** The heatmap showed the relationship among the risk grouping with the patient status, metastasis, EMT Score, T cell CD8, and monocyte infiltration abundance. **(C)** The comparison of EMTscore between the low-risk group and high-risk group. **(D)** The comparison of CD8 T-cell and monocytes’ infiltration between the low-risk group and high-risk group. **(E–G)** The survival curves of EMT score, T-cell CD8, and monocytes.

As mentioned above, the GPCRs related to OS-risk were highly enriched in immune receptor activity and Chemokine signaling pathway. Therefore, we attempted to investigate whether the risk grouping of the 10-GPCRs model was associated with the infiltration of immune cells. We used the CIBERSORTx to calculate the abundance of immune cells in each sample, by removing one outlier sample, and then, “limma” was used to analyze the difference infiltration of immune cells between the high-risk group and the low-risk group. We observed that CD8 T cells (P=0.00191) and monocytes (P=0.01735) were more aggregated in the low-risk group (**Figure 7A**). The heatmap illustrates the relationship between the risk grouping and the patient’s status, metastasis, EMT score, and the infiltration abundance of CD8 T-cells and monocytes (**Figure 7B**). Statistical analysis indicated the EMT score was not a significant difference between the low-risk group and high-risk group (**Figure 7C**), and the infiltration of CD8 T-cell and monocytes was significantly more aggregated in the low-risk group (**Figure 7D**). By survival analysis, we found that EMTscore could not predict the survival rate of OS patients (**Figure 7E**), while the infiltration abundance of CD8 T-cells could effectively predict the survival rate of patients (**Figure 7F**), which conforms to the logic that CD8 T-cells could kill tumor cells for treatment, but unfortunately monocytes could not effectively predict the survival rate (**Figure 7G**). All in all, monocytes and CD8 T-cells related to immune activation were more aggregated in the low-risk group, this might be why the risk score of the model could predict patients’ survival.

### The Expression of 10 GPCRs in OS Microenvironment

**Figure 8:**
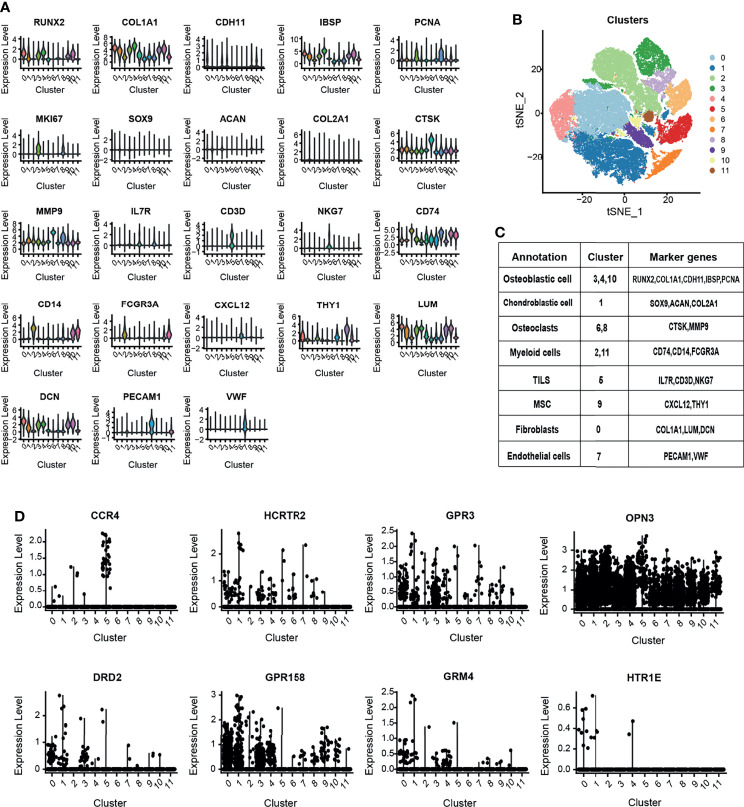
**(A)** The violin plot shows the expression level of canonical biomarkers in each cell population. **(B)** The tSNE plot shows the distribution of each cell population. **(C)** Use the canonical markers to annotate the 12 distinct clusters in OS. **(D)** OS single-cell atlas to identify the expression and distribution of the GPCRs in the OS micro-environment.

By analyzing the OS single-cell RNA-seq dataset, we obtained the OS atlas (**Figures 8A–C**), the atlas contains a total of 82,567 cells and 26,163 genes. The OS atlas was annotated into 11 cell subgroups and divided into immune cell subgroups and non-immune cell subgroups, among which the immune cell subgroups were the cluster 2, 5, 11, the non-immune cell subgroups were the cluster 0, 1, 3, 4, 6, 7, 8, 9, 10. Then mapping the expression of 10 GPCRs in the OS atlas (**Figure 8D**), the results showed that CCR4 was mainly expressed in tumor-infiltrating lymphocytes (TILs), and its positive-cell proportion (PCP) in the tumor micro-environment was about 0.046%. HCRTR2 was mainly expressed in non-immune cells, and its PCP was about 0.13%. GPR3 was mainly expressed in non-immune cells, and its PCP was about 1.36%. DRD2 was mainly expressed in non-immune cells, and its PCP was about 0.11%. GPR158 was mainly expressed in non-immune cells, and its PCP was about 1.05%. GRM4 was mainly expressed in non-immune cells, and its PCP was about 1.05%. OPN3 was generally expressed in various types of cells in the OS micro-environment, and its PCP was about 9.87%. HTR1E was mainly expressed in non-immune cells, and its PCP was about 0.02%. Among them, the expression of HTR1A and GPR144 could not be detected in this OS atlas, it might be caused by the low RNA capture rate at single-cell level. This result simply revealed the cellular expression context of the GPCRs.

### OS Targeted Drugs Based on the Model Signature

**Figure 9:**
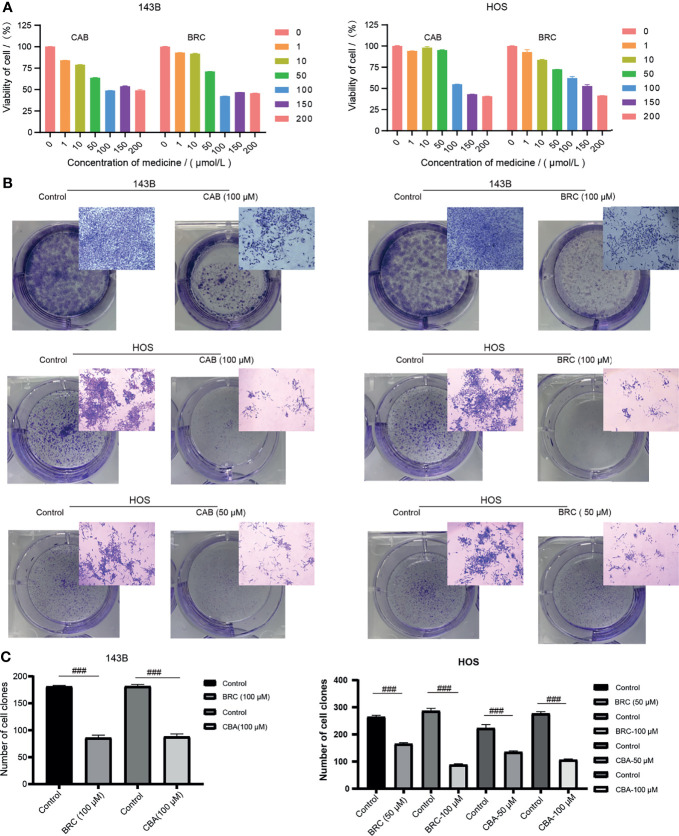
**(A)** Different concentrations of cabergoline and bromocriptine on the viability of 143B and HOS cell line, three repetitions. **(B)** The results of the colony formation experiment, including the well-plate image and microscope image, three repetitions. **(C)** Quantitative comparison of the number of the colony in the well-plate, “###” represents p < 0.001, n = 3. CAB, Cabergoline; BRC, bromocriptine.

The prognostic value of 10-GPCRs in the OS has been demonstrated above. Therefore, aimed at the 10 GPCRs as therapeutic targets may improve the treatment effect of the OS. Consistent with our research expectations, we found 3 targets with known drugs in 10GPCRs, they were CCR4, DRD2 and GRM4. Among them, CCR4 has a drug in the trial, DRD2 has 66 approved drugs and 32 trial drugs, GRM4 has 2 drugs in trials (21). Thus, DRD2 was the easiest target for drug repurposing, and due to the DRD2 was a protective factor, we hypothesize its agonist will give a better treatment effect on OS. Therefore, we used the agonists of DRD2 (cabergoline and bromocriptine) to test our hypothesis, and we found that cabergoline and bromocriptine did indeed have an inhibiting effect on OS by CCK8 cell viability experiment, where the half-inhibitory concentration (IC50) of cabergoline and bromocriptine in metastatic-OS (143B) were about 100 μM, the IC50 value of cabergoline in non-metastatic-OS (HOS) was about 100 μM, the IC50 value of bromocriptine in the HOS cells was about 150 μM, (**Figure 9A**). The colony formation experiment confirmed that cabergoline and bromocriptine could significantly inhibit the colony formation of 143B and HOS, which was characterized by smaller colony spots and fewer colonies (**Figure 9B**), the quantitative comparison between the experimental group and the control group showed that the drugs inhibitory effects were significant (**Figure 9C**). Here we provided an effective novel target with known drugs, this might increase new drug options for the treatment of OS.

## Discussion

According to cancer statistics in 2020, the 5-year survival rate of the OS is still only 67-69% (1). The conventional treatment for OS patients is surgical resection and neoadjuvant chemotherapy, but the clinical outcomes have been little improvement since the 1970s. Therefore, there is an urgent need for potential bio-targets to assess the risk of OS patients.

In this study, we obtained the expression profiles of GPCRs from the TARGET-OS database on the UCSC Xena platform. By using univariate Cox regression analysis, Lasso-Cox regression analysis, and multivariate Cox regression analysis, 10 GPCRs that were significantly related to OS risk were obtained. A prognostic model of OS based on the above 10 GPCRs was established. This model had high reliability (Kaplan–Meier curve’s p-value < 0.001, and AUC > 0.9), and high discriminatory ability in predicting survival rate. In the model, 6 GPCRs (CCR4, HCRTR2, GPR3, DRD2, HTR1A, GPR158) were highly associated with a low risk of OS (protective factor), and 4 GPCRs (GRM4, OPN3, GPR144, HTR1E) were highly associated with a high risk of OS (risk factors). Furthermore, we determined that the model and the metastasis could be used as independent risk prognostic factors through the joint analysis of univariate and multivariate. In addition, based on the Pearson correlation analysis between the metastasis and the expression level of 10-GPCRs, it was believed that the expression of DRD2 (P = 0.0031) was negatively correlated with the OS-metastasis, and the expression of HTR1E (P = 0.018) was positively correlated with OS-metastasis. We also found that the model could distinguish the samples with different immune cells, for example, the CD8 T-cells and monocytes were more aggregated in the low-risk group, and the infiltration ratio of CD8 T-cells was associated with a high survival rate of OS. The single-cell atlas was used to detect the cellular identity of 10-GPCRs in the OS micro-environment. Finally, in 10-GPCRs, we found that DRD2 was a target with approved drugs. By targeting DRD2, the agonists (bromocriptine and cabergoline) have the effect of inhibiting OS. Thus, we found targets with known drugs based on the risk model signature by using total GPCRs modeling for cancer prediction.

For protective factors, CCR4 was expressed in multiple subtypes of T cells, including effector CD8 T cells, Chemotaxis assay indicated that CCR4^+^ CD8 T cells could be recruited by CCL22 treatment (29), which was consistent with the finding that CCR4 was higher expressed in the low-risk group. Meanwhile, CD8 T cells were relatively higher infiltrated in the low-risk group. Both were correlated with a prolonger OS survival. In various cancer cell lines, HCRTR2 expression was down-regulated, which may initiate promoter hypermethylation (30, 31). After orexin receptor HCRTR2 was activated by orexins, apoptosis would be induced (32), indicating that the HCRTR2 had similar epigenetic characteristics to tumor suppressors. For GPR3, its agonists may render the breast cancer cells susceptible to cytotoxicity induced by cationic amphiphilic drugs, leading to lysosomal-dependent cell death (33), which showed the tumor-suppressive effect (34). By using the specific agonist, HTR1A can inhibit DNA synthesis (35), with lower expression in invasive cancer cells compared with non-invasive cancer cells (36). GPR158 has higher methylation level in esophageal squamous cell carcinoma compared with mucosa (37), which can induce apoptosis and is related to the high survival rate of glioma patients (38). For DRD2, it was considered as a tumor suppressor (39). It was reported that the DRD2 on cell membrane could exert anti-tumor effects by down-regulating eEF1A2 (40), while the eEF1A2 could promote the proliferation, migration, and invasion of OS cells by activating the Akt/mTOR signaling pathway (41), which implies that DRD2 has the anti-OS effect. It was also reported that the DRD2 agonists could exert anti-tumor effects through ROCK-mediated inactivation of Cofilin-1 or inhibiting EGFR/AKT/MMP-13 pathway (42, 43). It’s worth noting that the positive expression of Cofilin-1 in OS was related to clinical stage, distant metastasis, and tumor grade (44), and the high expression of EGFR in OS was related to high proliferative activity, metastatic potential, and poor prognosis (45), suggesting that DRD2 could be a drug target of OS. To summarize, the reports about the function of the protective factors were consistent with the view that high expression of the protective factors was associated with a higher survival rate of OS in this study.

For risk factors, our previous study showed that the high expression of GRM4 was associated with the lower survival rate of OS patients (46). For OPN3, its high expression was associated with a lower survival rate in lung adenocarcinoma and acral melanoma (47, 48). GPR144 (ADGRD2) was an important mediator in the hypoxic response of glioblastoma and had a significant tumor-promoting effect (49), associated with rheumatoid factor and osteosarcoma proliferation and invasion (50). For HTR1E, the activation of HTR1E could regulate the release of cytokines IL-6 and CXCL8 (51), which mediate osteosarcoma-lung interactions crucial to metastasis (52). The above report about the risk factors was consistent with our statistical results.

Bromocriptine and cabergoline are known drugs targeting DRD2 and are clinically used to treat pituitary adenomas and Parkinson’s disease. Treated for 24 h, the IC50 value of bromocriptine and cabergoline was about 100μM for some pituitary tumor cell lines (53), which is a similar lethal dose. Notably, bromocriptine can induct the expression level of DRD2 (54), which may upregulate the tumor suppressor (39). Besides, cabergoline and bromocriptine may also target other receptors, such as cabergoline can target 5HT(2A-C), D_3_, and 5HT(1A) (also call HTR1A, as a protective factor in this study) (55). Bromocriptine was reported to regulate amino acid biosynthesis and metabolism (56). This study may provide a novel insight into the drug therapy of OS.

## Conclusion

In summary, based on GDC TARGET and GPCRdb, we constructed an effective 10-GPCRS risk-score model (including CCR4, HCRTR2, GPR3, DRD2, HTR1A, GPR158, GRM4, OPN3, GPR144, HTR1E) to predict OS prognosis. The stability and accuracy of the model were evaluated by ROC curve. In addition, we found 3 GPCRs targets with known drugs in 10-GPCRs model, among which one target (DRD2) and its drugs (bromocriptine and cabergoline) were confirmed to inhibit the OS cells in this study. This study may provide reference for OS therapy in clinic.

## Data Availability Statement

Publicly available datasets were analyzed in this study, these data are included in the article material, further inquiries can be directed to the corresponding authors.

## Author Contributions

MT contributed to the conception/design, collection, and/or assembly of data, data analysis, interpretation, and manuscript writing. LZ, JZ, and MH contributed to the conception/design, supervision, and manuscript modification. SG contributed to the colony formation experiment, manuscript writing and discussion. XR contributed to the cell viability assay and colony formation experiment. All authors: final approval of the manuscript.

## Funding

This study was financially supported by the National Key R&D Program of China (2018YFC1105900), National Natural Science Fund of China (Grant No. 81972120), the Guangxi Science and Technology Base and Talent Special Project (Grant No. GuikeAD19254003), the Distinguished Young Scholars Program of Guangxi Medical University, the Youth Science Foundation of Guangxi Medical University(GXMUYSF202107), and the Scientific Research Project for Young Teachers of Guangxi-Collaborative Innovation Center for Biomedical (02406221008C).

## Conflict of Interest

The authors declare that the research was conducted in the absence of any commercial or financial relationships that could be construed as a potential conflict of interest.

## Publisher’s Note

All claims expressed in this article are solely those of the authors and do not necessarily represent those of their affiliated organizations, or those of the publisher, the editors and the reviewers. Any product that may be evaluated in this article, or claim that may be made by its manufacturer, is not guaranteed or endorsed by the publisher.
